# Distinct serum metabolomic signatures of multiparous and primiparous dairy cows switched from a moderate to high-grain diet during early lactation

**DOI:** 10.1007/s11306-020-01712-z

**Published:** 2020-09-09

**Authors:** C. Pacífico, A. Stauder, N. Reisinger, H. E. Schwartz-Zimmermann, Q. Zebeli

**Affiliations:** 1 Christian Doppler Laboratory for Innovative Gut Health Concepts of Livestock, Institute of Animal Nutrition and Functional Plant Compounds, Department for Farm Animals and Veterinary Public Health, University of Veterinary Medicine, Vienna, Austria; 2 Christian Doppler Laboratory for Innovative Gut Health Concepts of Livestock, Institute of Bioanalytics and Agro-Metabolomics, Department of Agrobiotechnology (IFA-Tulln), University of Natural Resources and Life Sciences, Vienna, Austria; 3 BIOMIN Research Center, BIOMIN Holding GmbH, Tulln, Austria

**Keywords:** Ruminal acidosis, Parity, Dairy cow, Early lactation, Targeted metabolomics

## Abstract

**Introduction:**

Feeding of high-grain diets is common in cows during early lactation, but increases the odds of metabolic derailments, which can likely be detected as undesirable shifts in the serum metabolome signature.

**Objectives:**

The present study aimed to identify the metabolic signatures of the serum metabolome of early lactation dairy cows switched from a moderate to a high-grain diet.

**Methods:**

Targeted ESI-LC-MS/MS-based metabolomics was used to characterize metabolic alterations in the serum of early lactation multiparous (MP, n = 16) and primiparous (PP, n = 8) Simmental cows, according to parity and feeding phase. Data were analysed using different data mining approaches.

**Results:**

Carnitine, acetylcarnitine, propionoylcarnitine, amino acid related compounds cis-4-hydroxyproline, trans-4-hydroxyproline, proline betaine, lysophosphatidylcholine PC a C16:1 and phosphatidylcholine PC ae C36:0 were identified as the key metabolites distinguishing MP from PP cows. A different serum metabolite composition during moderate and high-grain diet was also evident. Notably, cows fed high grain diet had higher serum concentrations of primary bile acids and triglycerides, but lower levels of conjugated bile acids and carboxylic acids during the first week in grain. Amino acids valine, cystine and taurine together with lysophosphatidylcholine PC a C26:0 and several phosphatidylcholines were classified as important features for cluster separation.

**Conclusions:**

Our study greatly expands earlier observations on dietary effects on serum metabolome composition of cows. The altered metabolomic fingerprints clearly distinguishable by diet and cow parity hold potential to be used as early diagnostic tools for cows experiencing grain-induced metabolic disturbances.

**Electronic supplementary material:**

The online version of this article (10.1007/s11306-020-01712-z) contains supplementary material, which is available to authorized users.

## Introduction

In dairy cows, early lactation represents both the peak of milk production and the highest requirements for energy and nutrients. Therefore, cows are commonly fed large amounts of concentrates during early lactation (Humer et al. 2016a; Nielsen et al. 2003; Saleem et al. 2012). However, despite providing large amounts of metabolizable energy, feeding of high amounts of starchy concentrates contributes to the high incidence of metabolic diseases in cattle (Ametaj et al. 2010). This is because starchy concentrates are rapidly degraded in the rumen, producing large amounts of short-chain fatty acids that often lead to the acidification of the ruminal fluid (Zebeli et al. 2008). Indeed, rumen acidosis has become a common digestive disorder in cattle production (Valente et al. 2017). It is characterized by a decrease of the ruminal pH, affecting both the composition and metabolic capability of the rumen microbiota, increasing the risks of microbial dysbiosis as well as “leaky gut” (Fernando et al. 2010; Khafipour et al. 2009) where the permeability of the rumen and intestinal epithelium increases as a result of this microbial dysbiosis (Aschenbach and Gäbel 2000; Emmanuel et al. 2007). Moreover, microbe-derived toxic compounds produced during dysbiosis are then translocated into systemic circulation. This chain of events culminates in a systemic inflammation and metabolic impairment, originating diseases such as fatty liver, rumen mucosal damage and/or liver abscess, displaced abomasum and laminitis (Plaizier et al. 2008). The susceptibility of dairy cows to acidosis appears to be highest for cows in early lactation (Penner et al. 2007; Oetzel 2003). In addition, clinical observation data have previously shown that PP cows have a higher risk of ruminal acidosis postpartum than MP cows (Krause and Oetzel 2006). Other reports suggest that although MP cows have shown a higher feed intake compared to PP cows, higher milk production potential makes them more susceptible to a stronger negative energy balance and related metabolic disturbances than naiver cows (Humer et al. 2016a).

Feeding of high amounts of rapidly fermentable carbohydrates was previously implied in changes of the patterns of plasma metabolites in cows (Ametaj et al. 2009). Single plasma metabolites such as non-esterified fatty acids, triglycerides, beta-hydroxybutyrate, cholesterol or glucose are often used to monitor the metabolic profile and health status of dairy cows (Guo et al. 2013). Yet, the alterations of single metabolites are not specific enough and it is difficult to establish a causal relationship of a specific derailment by singe metabolites. Due to the recent developments in high throughput metabolomics analysis, it is now possible to detect multiple classes of metabolites that reflect broader metabolic shifts which allows a more comprehensive assessment of the mechanisms behind metabolic impairment (Hailemariam et al. 2014a,b). This is particularly relevant for early diagnosis and understand the aetiology of metabolic derailments that originate from impairment of the gut microbiota due to malnutrition, which in turn may lead to systemic disorders. Therefore, changes of the metabolomic profile might improve both diagnostic tools and also help understand the causal relationship between malnutrition, dysbiosis and the derailments. Ametaj et al. (2010) were one of the first to use untargeted metabolomics approaches to describe metabolomic changes in the rumen in response grain-rich diets in cattle. Previously, we have used dry cows as a model to assess the relationship between grain-rich feeding and metabolic derailments in the rumen (Humer et al. 2018a). In that study, we reported a significant increase of lipopolysaccharide, biogenic amines and acidosis risk in the rumen, which went along with systemic inflammation and a decrease in phosphatidylcholines, lysophosphatidylcholines, sphingomyelins, and several amino acids in the blood of cows fed 65% concentrate in the diet. However, data that relate metabolomic changes with the increase of grain amount in the diet of early lactating dairy cows of different parities is still lacking in the literature. Changes of the serum metabolomic profile of dairy cows may reflect the ruminal acidotic condition of the cows in response to a high-grain challenge, and the parity may also play a role on how the animals react to it, helping to identify key metabolic features that distinguish cows experiencing rumen acidosis. Our main hypothesis was that high-grain challenge will lead to an impaired energy/lipid metabolism and systemic inflammation which will be reflected in the serum metabolome, and that this effect will be greater in PP cows.

## Materials and methods

### Animals, feeding and study design

This experiment was part of a larger study and detailed information about cows, feeding, and experimental design, ethical committee protocols as well as results with respect to feed intake, sorting, chewing activity, milk components, and rumen acidosis index have been reported in our companion paper (Stauder et al. 2020). Briefly, twenty-four early-lactating Simmental cows were used. The group consisted of 8 PP and 16 MP (average lactation number = 4.1 ± 1.9; mean ± SD) early lactation Simmental cows milked twice a day and housed in a loose-stall barn equipped with deep litter straw cubicles and a deep-bedded pack-area (10 × 8 m). Cows were kept at the research dairy farm of Vetmeduni Vienna (Pottenstein, Austria) and adaptation to the experimental barn area and the individual feeders took place for approximately 1 week before starting the trial. The animals weighted 737 ± 90 kg and were 50 ± 22 days in milk (DIM) at the start of the trial. A summary of dry matter intake, milk yield, and the rumen acidosis index of the PP and MP cows is given in Table S1. Details regarding recording of dry matter intake, milk yield and the analyses of milk composition as well as these results are reported in Stauder et al. (2020). The feeding model of this study, aiming to induce a rumen acidotic challenge by increasing the grain level in the diet, is similar to a previous model established by our team (Kröger et al. 2019). This feeding challenge model consisted by feeding the cows first fed a moderate-grain diet (M diet; 60% roughage and 40% concentrate—on dry matter basis) for 2 weeks, though providing enough energy and nutrients for cows around 50 days in milk and producing around 35 kg milk/day with 4% fat and 3.6% protein. To induce the rumen acidotic challenge, cows were then switched to a high-grain diet (H diet; 40% roughage and 60% concentrate) for four weeks resulting in 32% starch and 14% physically effective fiber, which are known to induce a rumen acidotic challenge (Zebeli et al. 2012). This feeding model has been successful to induce a mild subacute rumen acidosis challenge in cows, while avoiding carry over effects of high grain feeding, which last for at least 3 weeks (Qumar et al. 2017). Daily diet was fed as a total mixed ration and consisted of high quality forages such as grass silage, corn silage, and a concentrate mixture. Diet ingredients and chemical composition are given in Table S2, whereas details of feed sampling and analyses are given in Stauder et al. (2020).

### Blood sampling

Blood samples were collected before the morning feeding from the jugular vein of all animals on the last day of the week 2 of M-diet feeding, as well as on the last day of the week 1 (H-wk1) and on the last day of the week 4 (H-wk4) of the H-diet feeding period. This sampling design allowed us to contrast the M feeding with both a short-term (H-wk1) and long-term (H-wk4) challenge of high-grain feeding. Samples were taken using vacutainer tubes (9 ml, Vacuette, Greiner Bio-One, Kremsmuenster, Austria). Samples were allowed to clot at 25 °C for 2 h and all tubes were then centrifuged at 2000×*g* at 4 °C for 15 min (Eppendorf, Centrifuge 5804 R) to separate serum. Serum was pipetted into 2 mL tubes (Eppendorf) and stored at − 80 °C for further analysis.

### Metabolome profiling

Determination of the serum metabolome was carried out using a targeted metabolomics approach based on the Biocrates MxP® Quant 500 kit (Biocrates Life Sciences AG, Innsbruck, Austria). Ten μL aliquots of the serum samples were processed according to the manufacturer’s instructions. Analysis of sample extracts as well as of reference standards and quality controls (provided by the manufacturer) was carried out by ultra-high performance liquid chromatography (uHPLC) and flow injection analysis (FIA), both coupled to tandem mass spectrometry. An Agilent 1290 series UHPLC system coupled to a 6500 + QTrap mass spectrometer equipped with an Ion-Drive Turbo V® ESI source (both Sciex, Foster City, CA, USA) was used for the analysis. Chromatographic and mass spectrometric parameters were set as indicated by the manufacturer of the kit. Data analysis was carried out in Analyst 1.6.3 (Sciex) for LC–MS/MS data and in the Biocrates MetIDQ software for FIA-MS/MS data. The Biocrates MxP® Quant 500 kit can be used for analysis of up to 630 serum metabolites from 26 compound classes of widely different structure and polarity. Compound classes include lipids like acylcarnitines (Cx:y), hydroxylacylcarnitines [C(OH)x:y] and dicarboxylacylcarnitines (Cx:y-DC), lysophosphatidylcholines, phosphatidylcholines, sphingomyelins (SMx:y) and sphingomyelin derivatives [SM(OH)x:y], ceramides and derivatives (cer-, hexcer-, hex2cer-, and hex3cer-), cholesteryl esters, diglycerides and triglycerides, which are all measured by FIA-MS/MS, as well as amino acids, amino acid related compounds, bile acids, biogenic amines, the sum of hexoses (H1), p-cresol sulfate, carboxylic acids, fatty acids, hormones and related metabolites (abscisic acid, cortisol, cortisone, dehydroepiandrosterone sulfate; DHEAS), indoles and derivatives (indole, 3-indoleacetic acid, 3-indolepropionic acid, indoxyl sulfate), xanthine and hypoxanthine, choline, trigonelline and trimethylamine N-oxide (TMAO), which are determined by uHPLC-MS/MS.

### Statistical analysis

After a quality check, a total of 393 metabolites with < 50% missing values and compound concentration data of 72 samples were considered for further analysis. A repeated-measures analysis was conducted was performed using the MIXED procedure of SAS (version 9.3; SAS Inst. Inc., Cary, NC, USA). Variables were first tested for normality using the Shapiro–Wilk test. All statistical models were performed with the feeding phase (M, H-wk1, H-wk4), parity (PP, MP) and their interaction (feeding phase x parity) as fixed effects. To account for repeated measurements over time of the same animal within a feeding group, a first-order autoregressive variance–covariance structure was used, according to Bayesian information criterion. Cows nested within the experimental run and group were considered as random effects. Means were reported as least-squares means ± standard error of the mean (SEM). Comparisons among the least squares means were performed with the pdiff option. Statistical significance was declared at *P* ≤ 0.05 and as a trend towards significance when 0.05 < *P* ≤ 0.10. The *P*-values of all models regarding parity differences and comparisons among the least squares means between feeding phases were adjusted using the false discovery rate (FDR).

To find patterns in the data and significant features, multivariate analysis was performed using MetaboAnalyst 4.0 software (https://www.metaboanalyst.ca; (Chong et al. 2018)). The dataset consisted of 72 samples that were further analyzed in regards to parity (PP n = 24; MP n = 48) and feeding (M, H-wk1 + H-wk4). All missing values, zeros and negative values were replaced by the half of the minimum positive value in the original data. Feature filtering was performed based on interquantile range and row-wise normalization was using the primiparous group and M as reference. Data was mean centered and divided by the standard deviation of each variable and analyzed by multivariate statistical analysis using principal component analysis (PCA) (Figs. S1 and S2), and orthogonal-orthogonal projections to latent structures discriminant analysis (OPLS-DA). S-plots were used to reveal which variables (i.e., serum metabolites) were most responsible for the variation within the dataset combining the covariance and the correlation (p(corr)) loading profile. Hierarchical clustering analysis (HCA) with Euclidean distance measures and an average linkage method was also performed to explore the presence of clustering patterns among the serum metabolites according to diet, and the expression patters were visualized in a heatmap.

To conduct metabolite set enrichment analysis (MSEA), data were mapped according to the Human Metabolome Database (HMDB, www.hmdb.ca). Quantitative Enrichment Analysis (QEA) was performed for all metabolite classes, except triglycerides, and all individual metabolites that could match HMDB. Metabolites were evaluated according to their functional significance regarding parity (MP vs. PP) and diet (M-diet vs. H-diet) and the quantitative enrichment analysis was performed using the *globaltest* package to estimate a Q-statistic for each metabolite set (Goeman et al. 2004). Pathway analysis was further carried out to identify the most significant pathways responsible for the differences between groups using the Kyoto Encyclopedia of Genes and Genomes (KEGG). The pathway library for cow *(Bos taurus*) was selected for pathway enrichment analysis based on Globaltest and the node importance measure for topological analysis chosen was relative betweenness centrality.

## Results

### Differences in serum metabolome of MP and PP cows

**Fig. 1:**
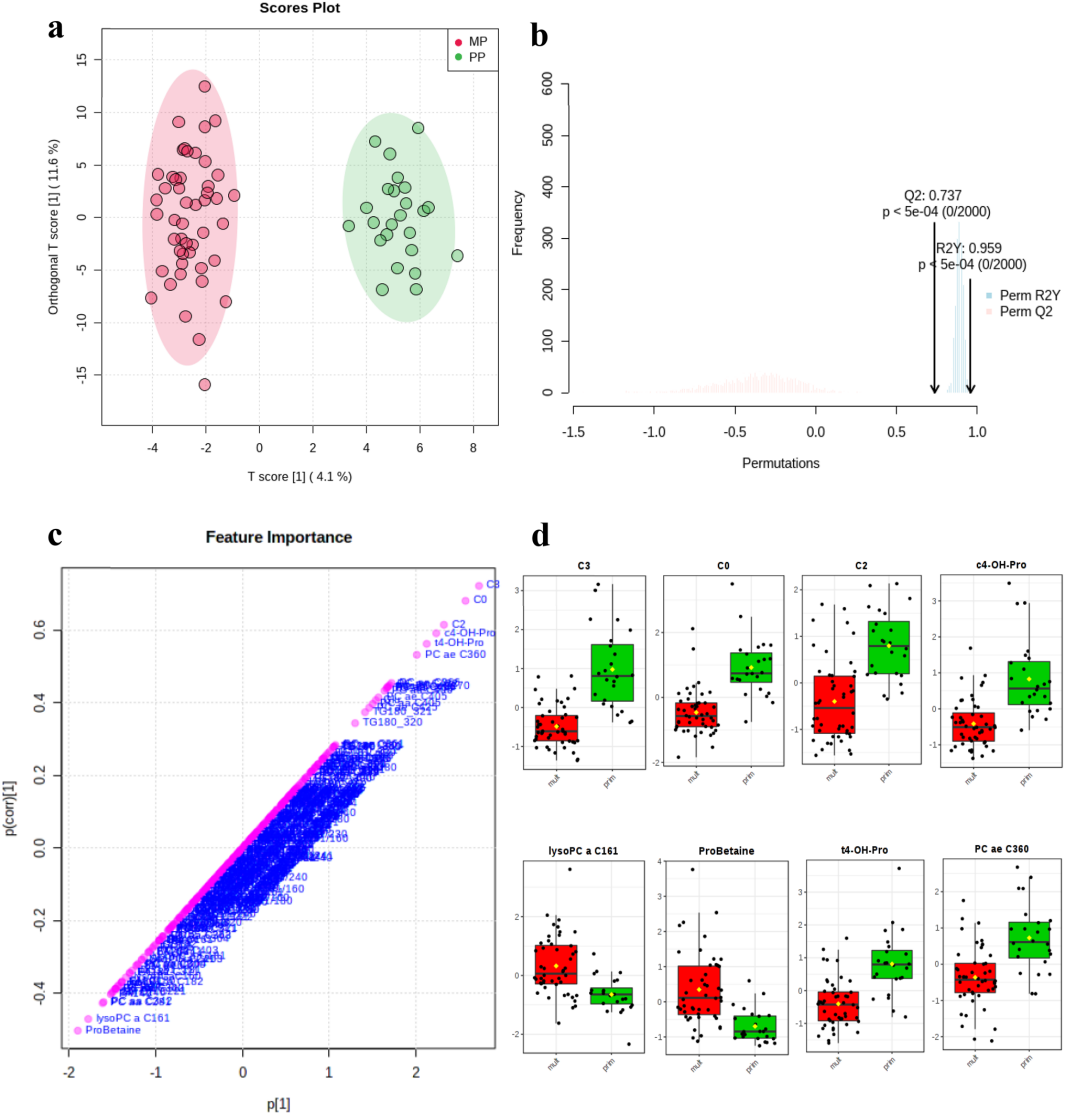
Orthogonal-orthogonal projections to latent structures discriminant analysis (OPLS-DA) showing the cluster separation between MP and PP cows (**a**). Model validation was performed using a permutation test (**b**). Feature importance was ranked in an S-plot, combining both the covariance and the correlation loading profile (**c**) and identified the top 8 metabolites that were relevant for group separation (**d**). The box and whisker plots summarize the normalized concentration values for carnitine (C0), acetylcarnitine (C2), propionoylcarnitine (C3), *cis*-4-hydroxyproline (c4-OH-Pro)*, trans*-4-hydroxyproline (t4-OH-Pro), proline betaine (ProBetaine), lysophosphatidylcholine C16:1 (lyso PC a C16:1) and phosphatidylcholine C36:0 (PC ae C36:0)

Global serum samples from PP (n = 24) and MP (n = 48) cows were analysed in this study to identify the main metabolic features differing between the two parity groups during early lactation and high-grain diet. PCA (Fig. S1) and OPLS-DA (Fig. 1a) were used as clustering tools to identify the metabolites contributing to the discrimination between the parity groups. The cross validation of the OPLS-DA model (Fig. 1b) revealed a Q^2^ value with significant cross-validated values between parity groups (Q^2^ = 0.737, R^2^Y = 0.959 and permutation test *P*-value < 0.00 for 2000 permutations). The OPLS-DA feature significance S-plot (Fig. 1c) recognized the carnitine, acetylcarnitine, propionoylcarnitine and the amino acid related compounds *cis*-4-hydroxyproline*, trans*-4-hydroxyproline and proline betaine as the most important variables for cluster separation. The lysophosphatidylcholine lyso PC a C16:1 and phosphatidylcholine PC ae C36:0 were also identified as key features in this separation (Fig. 1d).

In order to get a deeper understanding of the differences in the serum metabolomic profile between cows of different parities fed various diets, a linear mixed model was conducted to identify further metabolites involved (Tables 1, 2 and 3). Parity was shown to affect significantly the serum levels of acylcarnitines, amino acids and amino acid-related compounds, deoxycholic acid and lipids such as sphingomyelins, fatty acids, triglycerides, cholesteryl esters, lysophosphatidylcholines and phosphatidylcholines (Tables 1, 2 and 3). PP cows had overall 40% more acylcarnitines than MP cows, particularly C0 (+ 49%, *P* < 0.01), C3 (+ 63%, *P* < 0.01), C4 (+ 34%, *P* < 0.01) and C2 (+ 28%, *P* < 0.01). Amino acid glycine (P = 0.04) was more abundant in MP cows; glutamate, inversely, was 21% higher in PP cows (*P* = 0.02). The amino acid-related metabolites betaine (*P* < 0.01) and proline betaine (*P* < 0.01) were 57% and 51% higher in MP cows when compared to PP cows. An interaction between feeding phase and parity was found significant for the amino acid proline, particularly in H-wk4, where PP cows had less proline than MP cows in equal circumstances (*P* = 0.01). The opposite was reported for *cis*-4-hydroxyproline (*P* < 0.01) and *trans*-4-hydroxyproline (*P* < 0.01) which were more abundant in PP cows. A higher serum accumulation of bile acids was evident in MP cows, particularly deoxycholic acid (*P* = 0.01) and cholic acid (*P* = 0.04), while chenodeoxycholic acid (*P* = 0.06) exhibited a trend of increase.

**Table 1:** Concentrations (μM, unless otherwise stated) of acylcarnitines, carboxylic and bile acids, and indoles and derivatives in the serum of multiparous (MP) and primiparous (PP) cows fed moderate (M) or high grain level for 1 (H-wk1) or 4 (H-wk4) weeks

Metabolite	Parity		Phase				*P*-value			
	MP	PP	M	H-wk1	H-wk4	SEM	Parity	*FDR*	Phase	Phase × parity
*Acylcarnitines*										
C0	2.84	4.23	3.06^c^	3.40^b^	4.14^a^	0.39	< 0.01	< *0.01*	< 0.01	0.13
C2	0.71	0.91	0.84^y^	0.75^z^	0.85^y^	0.05	< 0.01	*0.00*	0.06	0.87
C3	0.31	0.50	0.36^b^	0.45^a^	0.40^a,b^	0.03	< 0.01	< *0.01*	0.01	0.54
C4	0.08	0.11	0.09	0.10	0.10	0.01	< 0.01	*0.00*	0.31	0.96
C5	0.10	0.12	0.10^a,b^	0.10^b^	0.12^a^	0.01	0.02	*0.08*	0.08	0.13
C10	0.10	0.10	0.10^b^	0.09^b^	0.10^a^	0.00	0.34	*0.46*	0.00	0.67
C10:1	0.06	0.06	0.06^b^	0.06^a,b^	0.07^a^	0.00	0.35	*0.56*	0.01	0.21
C10:2	0.26	0.23	0.24	0.25	0.24	0.01	0.01	*0.24*	0.74	0.98
C12	0.08	0.07	0.08	0.07	0.08	0.00	0.01	*0.06*	0.18	0.91
C12-DC	0.39	0.37	0.38	0.38	0.39	0.01	0.09	*0.32*	0.42	0.18
C12:1	0.04	0.04	0.04^b^	0.04^b^	0.05^a^	0.00	0.40	*0.55*	< 0.01	0.73
C14	0.04	0.04	0.03	0.04	0.04	0.00	0.08	*0.29*	0.21	0.37
C16	0.03	0.03	0.03	0.03	0.03	0.00	0.67	*0.86*	0.17	0.05
C16-OH	0.02	0.02	0.02	0.02	0.02	0.00	0.49	*0.77*	0.08	0.68
C16:1-OH	0.01	0.01	0.01	0.01	0.01	0.00	0.41	*0.70*	0.63	0.05
C16:2	0.01	0.01	0.01	0.01	0.01	0.00	0.05	*0.64*	0.74	0.83
C16:2-OH	0.01	0.01	0.01	0.01	0.01	0.00	0.58	*0.79*	0.06	0.13
C18:1	0.05	0.04	0.04^b^	0.05^a^	0.05^a^	0.00	0.01	*0.04*	0.00	0.38
*Carboxylic acids*										
Hippuric acid	40.6	44.3	51.6^a^	38.6^b^	37.1^b^	3.72	0.27	*0.46*	0.01	0.90
Succinic acid	3.21	3.06	2.98	3.35	3.07	0.11	0.23	*0.38*	0.05	0.28
Aconitic acid	2.77	3.14	2.61	3.16	3.09	0.28	0.28	*0.53*	0.09	0.95
Dodecanedioic acid	0.41	0.45	0.48	0.44	0.37	0.05	0.60	*0.74*	0.04	0.98
3-hydroxyglutaric acid	0.82	0.79	0.79	0.87	0.75	0.04	0.45	*0.66*	0.06	0.85
*Bile acids*										
Cholic acid	91.5	55.3	32.11^b^	111^a^	76.9^a^	18.3	0.031	*0.07*	0.00	0.58
Chenodeoxycholic acid	2.67	1.51	0.66^b^	3.12^a^	2.48^a^	0.66	0.06	*0.11*	< 0.01	0.378
Deoxycholic acid	3.98	2.02	1.40^b^	4.44^a^	3.17^a^	0.78	0.01	*0.04*	0.00	0.44
Taurocholic acid	5.04	4.40	6.03	4.17	3.96	1.04	0.97	*0.97*	0.08	0.27
Taurodeoxycholic acid	1.44	1.04	1.96^a^	1.02^b^	0.74^b^	0.36	0.98	*0.98*	0.00	0.20
Glycodeoxycholic acid	2.08	1.43	2.04	1.81	1.42	0.40	0.15	*0.26*	0.08	0.11
Glycocholic acid	9.73	7.57	10.8	8.16	7.02	1.78	0.33	*0.64*	0.06	0.09
*Indoles and derivatives*										
Indoxyl sulfate	2.20	2.36	2.05^z^	2.23^y,z^	2.56^y^	0.15	0.47	*0.63*	0.01	0.91

^ab^Indicate differences among LS means within phase at *P* ≤ 0.05 after FDR correction

^yz^Indicate differences among LS means within phase when *0.05* < *P ≤ 0.10* after FDR correction

**Table 2:** Concentrations of amino acids, biogenic amines, amino acid related compounds and cresols in the serum of (MP) and primiparous (PP) cows fed moderate (M) or high grain level for 1 (H-wk1) or 4 (H-wk4) weeks

Metabolite	Parity		Phase				*P*-value			
	MP	PP	M	H-wk1	H-wk4	SEM	Parity	*FDR*	Phase	Phase × Parity
*Amino acids*										
Proline	98.4	92.5	90.6	96.57	99.2	3.99	0.18	*0.31*	0.12	0.01
Cysteine	52.4	46.7	43.6^c^	50.4^b^	54.6^a^	2.61	0.06	*0.10*	< 0.01	0.99
Glutamate	41.6	50.3	45.9^a,b^	49.6^a^	42.5^b^	2.36	0.02	*0.05*	0.01	0.85
Histidine	57.9	52.8	46.9^b^	57.9^a^	61.4^a^	6.08	0.26	*0.42*	0.00	0.14
Isoleucine	106	115	101^b^	109^a,b^	121^a^	6.32	0.07	*0.19*	0.01	0.50
Leucine	115	119	105^b^	113^b^	133^a^	5.78	0.52	*0.66*	< 0.01	0.48
Lysine	87.3	91.4	82.5^z^	87.3^y,z^	98.3^y^	5.12	0.45	*0.53*	0.03	0.55
Methionine	28.6	30.5	28.0	28.6	32.0	1.31	0.26	*0.55*	0.09	0.37
Glycine	375	320	348	360	335	32.5	0.04	*0.27*	0.68	0.26
Serine	130	113	124	121	119	7.17	0.05	*0.44*	0.88	0.28
Tryptophan	56.9	53.7	52.8	54.7	58.4	2.06	0.36	*0.53*	0.02	0.88
Valine	211	229	193^c^	221^b^	247^a^	8.51	0.11	*0.16*	< 0.01	0.15
Arginine	82.4	88.0	77.8	83.3	94.6	5.87	0.24	*0.50*	0.10	0.85
Threonine	106	105	107	103	105	6.06	0.91	*0.96*	0.71	0.09
*Biogenic amines*										
Histamine	0.24	0.24	0.24	0.24	0.24	0.00	0.46	*0.62*	0.03	0.05
Beta-Alanine	14.0	14.6	14.6	13.8	14.6	0.56	0.53	*0.73*	0.25	0.02
Putrescine	0.08	0.08	0.08	0.08	0.08	0.01	0.77	*0.85*	0.73	0.06
*Amino acid related*										
5-Aminovaleric acid	2.29	1.47	1.38^b^	2.63^a^	1.62^b^	0.41	0.01	*0.02*	< 0.01	0.32
1-Methylhistidine	4.20	3.73	4.15^a^	3.40^b^	4.35^a^	0.22	0.21	*0.36*	< 0.01	0.23
Anserine	0.49	0.40	0.46	0.43	0.44	0.46	0.04	*0.17*	0.46	0.22
Betaine	20.4	8.82	16.6	13.8	13.4	4.11	< 0.01	*0.00*	0.12	0.54
α-Aminobutyric acid	4.23	3.64	4.63^a,y^	3.37^b^	3.81^z^	0.39	0.24	*0.38*	< 0.01	0.11
α-Aminoadipic acid	2.70	2.29	2.24^z^	2.45	2.79^y^	0.19	0.18	*0.31*	0.02	0.62
Carnosine	14.9	15.5	14.2^b^	13.4^b^	18.0^a^	1.31	0.74	*0.88*	0.01	0.56
Cystine	17.8	15.5	13.4^b^	17.5^a,z^	19.1^a,y^	1.40	0.15	*0.21*	< 0.01	0.63
Homoarginine	1.44	1.67	1.29^b^	1.55^a,b^	1.8^a^	0.12	0.07	*0.14*	0.00	0.98
Ornithine	50.3	49.2	42.0^b^	51.9^a^	55.4^a^	3.60	0.91	*0.91*	< 0.01	0.77
Phenylacetylglycine	21.0	23.4	18.7^b^	26.8^a^	21.06^a,b^	2.76	0.27	*0.41*	0.01	0.66
*cis*-4-Hydroxyproline	11.3	17.3	16.3^a^	13.4^b^	13.2^b^	0.77	< 0.01	< 0.01	< 0.01	0.36
Proline betaine	1.20	0.59	0.89	0.92	0.86	0.11	< 0.01	*0.00*	0.69	0.42
Sarcosine	2.79	3.69	3.09	2.96	3.67	0.32	0.02	*0.17*	0.75	0.43
Taurine	59.2	55.6	48.1^b^	58.6^a^	65.5^a^	3.78	0.35	*0.42*	< 0.01	0.61
*trans*-4-Hydroxyproline	11.3	15.9	15.8^a^	12.5^b^	12.6^b^	0.54	< 0.01	< *0.01*	< 0.01	0.52
3-Methylhistidine	5.37	4.78	5.23	5.13	4.87	0.38	0.30	*0.52*	0.28	0.04
*Cresols*										
p-Cresol sulfate	46.4	50.4	42.9^b^	49.9^a,b^	52.5^a^	3.65	0.47	*0.65*	0.01	0.67

^ab^Indicate differences among LS means within phase at *P* ≤ 0.05 after FDR correction

^yz^Indicate differences among LS means within phase when *0.05* < *P ≤ 0.10* after FDR correction

**Table 3:** Concentrations of sphingomyelins, fatty acids, diglycerides, triglycerides, ceramides and derivatives, lysophosphatidylcholines and phosphatidylcholines in the serum of multiparous (MP) and primiparous (PP) cows fed moderate (M) or high grain level for 1 (H-wk1) or 4 (H-wk4) weeks

Metabolite	Parity		Phase				*P*-value			
	MP	PP	M	H-wk1	H-wk4	SEM	Parity	*FDR*	Phase	Phase x parity
*Sphingomyelins*										
SM (OH) C14:1	20.4	20.6	18.7^b^	19.8^b^	23.2^a^	0.80	0.85	*0.90*	< 0.01	0.79
SM (OH) C16:1	12.5	13.3	11.4^c^	12.6^b^	14.9^a^	0.64	0.43	*0.51*	< 0.01	0.93
SM (OH) C22:1	13.5	11.8	12.8	12.4	12.8	0.48	0.03	*0.16*	0.73	0.08
SM(OH) C24:1	1.67	1.44	1.53	1.52	1.62	0.09	0.04	*0.16*	0.28	0.38
SM C16:0	135	121	125	126	133	4.73	0.08	*0.23*	0.30	0.38
SM C16:1	14.4	13.3	12.8^b^	13.7^z^	15.0^a,y^	0.49	0.18	*0.25*	< 0.01	0.65
SM C18:0	12.3	11.0	11.8	11.4	11.7	0.37	0.04	*0.22*	0.70	0.29
SM C18:1	4.38	4.43	4.11	4.29	4.81	0.25	0.86	*0.91*	0.02	0.57
SM C24:0	31.1	27.1	31.5^a^	29.0^a,b^	27.0^b^	1.07	0.00	*0.02*	0.01	0.84
SM C24:1	5.53	4.50	5.66^a^	4.85^a,b^	4.53^b^	0.38	0.02	*0.04*	0.02	0.42
*Fatty acids*										
Myristic acid	160	106	141	119	141	18.9	0.00	*0.03*	0.32	0.15
Octadecenoic acid	46.3	31.5	47.9	33.8	35.2	5.65	0.01	*0.07*	0.08	0.61
Octadecadienoic acid	57.6	40.8	49.2	47.0	51.3	4.14	0.01	*0.07*	0.54	0.78
Eicosenoic acid	1.08	0.75	1.02	0.89	0.85	0.12	0.00	*0.02*	0.40	0.93
Eicosadienoic acid	0.44	0.32	0.38	0.35	0.42	0.05	0.03	*0.24*	0.32	0.93
Eicosatrienoic acid	1.16	0.86	0.89^z^	0.96^y,z^	1.18^y^	0.10	0.04	*0.10*	0.06	0.53
Palmitic acid	307	289	314	294	286	9.66	0.06	*0.14*	0.07	0.32
Stearic acid	305	288	310	295	285	285	0.07	*0.21*	0.17	0.10
*Triglycerides*										
TG(14:0_36:2)	0.68	0.62	0.62	0.69	0.63	0.05	0.26	*0.41*	0.46	0.01
TG(16:0_32:2)	0.74	0.65	0.69	0.68	0.69	0.04	0.07	*0.82*	0.95	0.73
TG(16:0_35:2)	0.54	0.58	0.50	0.60	0.58	0.05	0.41	*0.55*	0.03	0.04
TG(16:0_36:3)	2.55	2.35	2.28	2.55	2.52	0.17	0.22	*0.51*	0.04	0.24
TG(16:0_36:4)	1.69	1.64	1.56	1.72	1.71	0.08	0.52	*0.82*	0.07	0.70
TG(16:0_38:1)	0.84	0.87	0.93	0.90	0.74	0.07	0.57	*0.90*	0.05	0.40
TG(16:0_38:7)	0.85	0.93	0.96	0.92	0.79	0.06	0.23	*0.49*	0.07	0.46
TG(16:1_30:1)	0.74	0.63	0.70	0.66	0.72	0.04	0.02	*0.16*	0.21	0.84
TG(16:1_36:2)	0.63	0.61	0.59	0.64	0.63	0.04	0.72	*0.82*	0.37	0.07
TG(16:1_36:3)	0.43	0.39	0.37	0.44	0.42	0.04	0.34	*0.80*	0.10	0.09
TG(17:0_32:1)	0.59	0.61	0.53	0.63	0.63	0.07	0.70	*0.80*	0.06	0.73
TG(17:0_34:1)	0.88	0.89	0.87	0.90	0.89	0.07	0.83	*0.92*	0.90	0.10
TG(18:0_30:0)	1.42	1.81	1.64	1.53	1.67	0.10	0.01	*0.06*	0.51	0.58
TG(18:0_32:0)	2.27	2.73	2.69	2.37	2.44	0.17	0.07	*0.25*	0.26	0.59
TG(18:0_32:1)	1.30	1.56	1.38	1.49	1.40	0.09	0.05	*0.25*	0.66	0.35
TG(18:0_32:2)	0.45	0.44	0.39	0.47	0.46	0.04	0.81	*0.85*	0.07	0.08
TG(18:0_38:7)	0.78	0.82	0.94	0.76	0.71	0.07	0.42	*0.61*	0.05	0.86
TG(18:1_26:0)	0.54	0.56	0.48	0.55	0.61	0.05	0.63	*0.75*	0.06	0.19
TG(18:1_30:0)	1.42	1.50	1.35	1.61	1.43	0.08	0.32	*0.55*	0.04	0.22
TG(18:1_32:1)	1.71	1.63	1.55	1.87	1.58	0.14	0.58	*0.74*	0.03	0.35
TG(18:1_33:2)	0.51	0.55	0.51	0.60	0.48	0.04	0.58	*0.81*	0.08	0.44
TG(18:1_34:1)	5.91	5.26	5.15	6.24	5.34	0.64	0.35	*0.51*	0.03	0.36
TG(18:1_34:2)	2.92	2.63	2.51^z^	3.03^y^	2.79^y,z^	0.26	0.29	*0.56*	0.01	0.52
TG(18:1_34:3)	0.66	0.55	0.58	0.67	0.57	0.04	0.01	*0.08*	0.11	0.19
TG(18:1_35:2)	0.52	0.48	0.46	0.58	0.46	0.08	0.47	0.68	0.02	0.70
TG(18:1_36:3)	5.37	4.93	4.91	5.37	5.18	0.31	0.06	0.30	0.15	0.96
TG(18:1_38:7)	0.60	0.59	0.60	0.63	0.54	0.06	0.21	*0.94*	0.90	0.10
TG(18:2_30:0)	0.61	0.56	0.52	0.62	0.62	0.03	0.37	*0.62*	0.02	0.75
TG(18:2_32:0)	1.13	1.17	1.02^b,z^	1.18^y^	1.25^a^	0.06	0.00	*0.67*	0.56	0.21
TG(18:2_32:1)	0.67	0.62	0.56^b^	0.73^a^	0.66^a^	0.06	0.41	*0.52*	0.00	0.74
TG(18:2_33:2)	0.34	0.27	0.29	0.32	0.31	0.04	0.05	*0.17*	0.22	0.12
TG(18:2_35:1)	0.44	0.40	0.39	0.50	0.40	0.06	0.76	*0.80*	0.08	0.55
TG(18:2_36:1)	1.55	1.48	1.40	1.65	1.51	0.12	0.32	*0.50*	0.05	0.15
TG(18:2_36:3)	7.06	6.71	6.73	6.84	7.08	0.23	0.05	*0.27*	0.18	0.76
TG(18:3_36:2)	0.43	0.36	0.34^z^	0.45^y^	0.40	0.08	0.14	*0.30*	0.03	0.17
TG(20:1_24:3)	0.28	0.25	0.31^a,b^	0.25^a^	0.24^b^	0.03	0.32	*0.47*	0.05	0.55
TG(20:1_31:0)	5.73	5.39	6.07^a^	5.69^a^	4.95^b^	0.19	0.10	*0.14*	< 0.01	0.95
TG(22:5_32:0)	0.40	0.49	0.44	0.43	0.45	0.05	0.07	*0.42*	0.38	0.33
*Cholesteryl esters*										
CE(14:0)	18.2	14.3	15.4^b^	14.7^b^	18.7^a^	1.18	0.02	*0.04*	< 0.01	0.95
CE(14:1)	0.91	0.60	0.76	0.76	0.76	0.09	0.00	*0.02*	0.99	0.20
CE(15:0)	38.1	40.1	40.9	35.5	40.9	2.24	0.56	*0.74*	0.03	0.99
CE(16:0)	39.3	37.5	42.1^a^	35.1^b^	37.9^a,b^	1.99	0.50	*0.71*	0.03	0.28
CE(16:1)	34.0	28.0	33.7^a,y^	29.9^b^	29.4^z^	1.73	0.03	*0.10*	0.12	0.04
CE(18:0)	2.19	2.19	2.55^a,y^	1.95^b^	2.07^z^	0.14	0.82	*0.82*	0.00	0.12
CE(18:1)	20.2	19.4	23.4^a,y^	19.3^z^	16.7^b,y^	1.35	0.77	*0.86*	< 0.01	0.01
CE(18:2)	840	725	712^b^	727^b^	908^a^	45.6	0.02	*0.04*	< 0.01	0.41
CE(18:3)	211	213	229^a^	194^b^	213^a,b^	9.38	0.88	*0.89*	0.02	0.47
CE(20:3)	8.71	7.91	7.05^b^	7.28^b^	10.6^a^	0.48	0.20	*0.29*	< 0.01	0.24
CE(20:4)	24.0	23.5	23.3^z^	21.4^b^	26.5^a,y^	1.66	0.66	*0.70*	< 0.01	0.02
CE(20:5)	25.5	27.5	28.7^a^	26.0^a,b^	24.4^b^	2.54	0.53	*0.67*	0.01	0.29
CE(22:5)	0.97	1.05	0.97^b^	0.91^b^	1.16^a^	0.09	0.48	*0.66*	< 0.01	0.14
CE(22:6)	2.12	2.58	2.42	2.24	2.37	0.15	0.01	*0.07*	0.65	0.09
*Ceramides and derivatives*										
Cer(d18:1/16:0)	0.24	0.24	0.27^a,y^	0.22^b^	0.24^z^	0.01	0.00	*0.94*	0.94	0.06
Cer(d18:1/18:0)	0.14	0.11	0.15^y^	0.12^y,z^	0.11^z^	0.03	0.08	*0.21*	0.07	0.14
Cer(d18:1/24:1)	0.23	0.19	0.23	0.19	0.20	0.01	0.09	*0.24*	0.10	0.39
HexCer(d18:1/24:1)	0.36	0.38	0.36	0.35	0.40	0.04	0.76	*0.81*	0.34	0.04
Hex2Cer(d18:1/18:0)	0.13	0.10	0.12	0.11	0.12	0.01	0.46	*0.32*	0.08	0.21
Hex2Cer(d18:1/24:0)	0.11	0.10	0.13^y^	0.11^y,z^	0.09^z^	0.02	0.41	*0.56*	0.04	0.14
Hex3Cer(d18:1/18:0)	0.24	0.21	0.22^a,b^	0.19^b^	0.26^a^	0.02	0.24	*0.35*	0.01	0.02
Diglycerides										
DG(16:0_20:0)	1.02	1.00	1.12^a,y^	0.97^z^	0.95^b^	0.06	0.84	*0.88*	0.09	0.41
DG(18:1_20:1)	0.10	0.09	0.09	0.09	0.10	0.01	0.08	*0.18*	0.30	0.00
*Lysophosphatidyl- cholines*										
lysoPC a C14:0	2.13	2.05	2.04	2.11	2.12	0.06	0.06	*0.36*	0.26	0.94
lysoPC a C16:0	10.5	9.25	9.72^z^	9.40^b^	10.5^a,y^	0.69	0.01	*0.03*	0.01	0.86
lysoPC a C16:1	0.89	0.73	0.84	0.77	0.81	0.05	< 0.01	*0.00*	0.33	0.04
lysoPC a C17:0	1.43	1.57	1.39^b^	1.46^b^	1.65^a^	0.09	0.09	*0.16*	< 0.01	0.82
lysoPC a C18:0	13.1	13.8	12.90	13.40	14.1	0.64	0.27	*0.46*	0.05	0.73
lysoPC a C18:1	9.59	8.48	9.85^a^	8.63^b^	8.65^b^	0.47	0.04	*0.09*	0.01	0.19
lysoPC a C18:2	17.9	14.9	15.5^a^	15.4^a^	18.2^b^	1.07	0.01	*0.03*	< 0.01	0.66
lysoPC a C20:3	2.71	2.38	2.41^a^	2.33^a^	2.89^b^	0.13	0.01	*0.17*	< 0.01	0.75
lysoPC a C20:4	1.49	1.45	1.52^a^	1.34^b^	1.55^a^	0.10	0.69	*0.77*	0.00	0.15
lysoPC a C24:0	0.45	0.46	0.52	0.44	0.42	0.03	0.53	*0.60*	0.02	0.30
lysoPC a C26:0	1.57	1.64	1.99^a^	1.49^b^	1.33^b^	0.22	0.69	*0.72*	< 0.01	0.80
lysoPC a C26:1	0.72	0.67	0.79^y^	0.65^z^	0.65^z^	0.09	0.51	*0.85*	0.02	0.40
lysoPC a C28:0	3.05	3.49	4.01^a^	3.10^b^	2.70^b^	0.46	0.22	*0.30*	< 0.01	0.70
*Phosphatidylcholines*										
PC ae C30:0	1.20	1.08	1.16^y^	1.08^b,z^	1.18^a^	0.05	0.06	*0.17*	0.04	0.63
PC ae C30:1	1.78	1.77	1.93^a^	1.65^b^	1.74^a,b^	0.09	0.95	*0.95*	0.00	0.90
PC ae C30:2	0.62	0.61	0.60	0.59	0.65	0.03	0.84	*0.88*	0.04	0.42
PC ae C32:1	5.51	5.05	5.55	4.94	5.35	0.24	0.18	*0.31*	0.05	0.45
PC ae C32:2	10.4	10.6	10.2	10.30	11.1	0.43	0.79	*0.95*	0.04	0.55
PC ae C34:0	3.67	3.89	3.91^a^	3.38^b^	4.06^a^	0.14	0.30	*0.41*	< 0.01	0.53
PC ae C34:2	28.5	24.9	25.2^b^	25.7^b^	29.0^a^	0.93	0.02	*0.04*	0.00	0.80
PC ae C34:3	41.7	37.7	32.9^c^	36.9^b^	49.2^a^	1.79	0.19	*0.22*	< 0.01	0.52
PC ae C36:0	2.58	3.03	2.95^a^	2.61^b^	2.85^a^	0.09	0.01	*0.02*	0.00	0.31
PC ae C36:2	39.7	38.6	34.6^b^	37.6^b^	45.2^a^	2.45	0.71	*0.75*	< 0.01	0.66
PC ae C36:3	11.8	11.8	11.4^z^	11.1^b^	12.9^a,y^	0.48	0.92	*0.96*	0.00	0.75
PC ae C36:4	9.31	7.65	7.51^b^	7.58^b^	10.4^a^	0.55	0.07	*0.12*	< 0.01	0.45
PC ae C36:5	5.22	4.79	4.44^b^	4.44^b^	6.15^a^	0.29	0.43	*0.58*	< 0.01	0.58
PC ae C38:0	2.24	2.44	2.53^a^	2.25^b^	2.25^b^	0.10	0.28	*0.42*	0.00	0.17
PC ae C38:1	3.98	4.34	4.46	4.14	3.87	0.21	0.34	*0.56*	0.06	0.61
PC ae C38:3	7.01	7.00	6.10^c^	6.82^b^	8.08^a^	0.34	0.98	*0.98*	< 0.01	0.78
PC ae C38:4	4.72	4.82	4.41^b^	4.43^b^	5.47^a^	0.26	0.77	*0.94*	< 0.01	1.00
PC ae C38:5	3.86	3.80	3.47^b^	3.47^b^	4.55^a^	0.23	0.93	*0.94*	< 0.01	0.84
PC ae C38:6	4.00	4.48	3.41^b^	3.90^b^	5.42^a^	0.33	0.30	*0.36*	< 0.01	0.33
PC ae C40:2	1.29	1.31	1.30^y,z^	1.24^z^	1.37^y^	0.05	0.84	*0.85*	0.02	0.51
PC ae C40:3	1.56	1.63	1.47^b^	1.60^a,b^	1.72^a^	0.09	0.71	*0.77*	0.01	0.89
PC ae C40:4	1.89	1.91	1.69^b^	1.83^b^	2.16^a^	0.12	0.91	*0.95*	< 0.01	0.92
PC ae C40:5	2.63	3.12	2.51^c^	2.80^b^	3.32^a^	0.27	0.16	*0.20*	< 0.01	0.75
PC ae C40:6	1.14	1.30	1.17	1.18	1.32	0.10	0.10	*0.30*	0.08	0.81
PC ae C42:0	0.91	0.89	0.94^y^	0.91^y,z^	0.85^z^	0.03	0.41	*0.64*	0.01	0.28
PC ae C42:1	0.44	0.42	0.46^a^	0.41^b^	0.41^b^	0.02	0.49	*0.58*	0.00	0.37
PC ae C42:2	0.33	0.38	0.37	0.34	0.36	0.01	0.03	*0.12*	0.18	0.88
PC ae C42:3	0.42	0.45	0.46^y^	0.42^z^	0.43^y,z^	0.02	0.39	*0.74*	0.03	0.05
PC ae C42:4	0.42	0.43	0.40^b^	0.41^b^	0.47^a^	0.02	0.56	*0.66*	< 0.01	0.85
PC ae C44:3	0.20	0.19	0.21^a^	0.20^a,b^	0.18^b^	0.01	0.73	*0.81*	0.02	0.94
PC aa C24:0	0.25	0.26	0.30^a^	0.25^b^	0.22^b^	0.01	0.70	*0.78*	< 0.01	0.91
PC aa C26:0	2.14	1.98	2.54^a^	1.91^b^	1.73^b^	0.25	0.39	*0.49*	< 0.01	0.98
PC aa C28:1	4.08	3.38	3.64^z^	3.54^b^	4.00^a,y^	0.20	0.01	*0.02*	0.02	0.28
PC aa C30:0	3.60	3.19	3.39^a,b^	3.22^b^	3.57^a^	0.15	0.04	*0.12*	0.01	0.61
PC aa C32:0	7.81	7.34	7.67^a,b^	6.97^b^	8.07^a^	0.28	0.26	*0.33*	0.01	0.65
PC aa C32:1	9.39	7.99	8.87	8.19	9.00	0.42	0.03	*0.11*	0.16	0.16
PC aa C32:2	10.9	10.4	10.7	10.0	11.1	0.51	0.58	*0.69*	0.04	0.15
PC aa C32:3	32.6	31.0	29.2^b^	29.3^b^	36.9^a^	1.75	0.59	*0.75*	< 0.01	0.90
PC aa C34:2	203	162	165^b^	174^b^	210^a^	8.37	0.00	*0.01*	< 0.01	0.78
PC aa C34:3	25.4	22.9	24.4^y^	21.7^b,z^	26.3^a^	0.93	0.08	*0.17*	< 0.01	0.91
PC aa C34:4	7.24	6.76	5.66^b^	6.04^b^	9.31^a^	0.37	0.43	*0.54*	< 0.01	0.78
PC aa C36:0	6.62	6.77	7.86^a^	6.42^b^	5.80^b^	0.26	0.69	*0.77*	< 0.01	0.37
PC aa C36:1	105	108	115^a^	106^a^	99.1^b^	4.99	0.00	*0.01*	< 0.01	0.78
PC aa C36:2	313	297	276^b^	300^b^	339^a^	11.8	0.38	*0.45*	< 0.01	0.76
PC aa C36:3	108	94.9	91.9^b,z^	101^a,y^	111^a,z^	5.11	0.11	*0.17*	< 0.01	0.55
PC aa C36:4	29.0	24.4	24.3^b^	25.2^b^	30.5^a^	1.57	0.03	*0.05*	< 0.01	0.56
PC aa C36:5	6.55	6.22	6.14^b^	5.83^b^	7.17^a^	0.36	0.58	*0.65*	< 0.01	0.71
PC aa C36:6	2.69	3.34	2.70^b^	2.89^b^	3.45^a^	0.18	0.06	*0.10*	< 0.01	0.50
PC aa C38:0	3.55	3.96	4.56^a^	3.58^b,z^	3.13^b,y^	0.19	0.08	*0.12*	< 0.01	0.71
PC aa C38:1	5.89	5.60	7.60^a^	6.01^b^	3.63^c^	0.38	0.61	*0.65*	< 0.01	0.60
PC aa C38:3	61.4	60.7	53.9^b,z^	60.0^b,y^	69.3^a^	3.38	0.70	*0.74*	< 0.01	0.74
PC aa C38:4	38.6	38.3	35.3^b^	36.9^b^	43.2^a^	2.17	0.77	*0.82*	< 0.01	0.36
PC aa C38:5	19.1	19.6	18.1	19.0	21.0	1.47	0.81	*0.91*	0.10	0.79
PC aa C38:6	3.56	3.72	3.43	3.54	3.95	0.28	0.59	*0.83*	0.06	0.71
PC aa C40:4	12.0	10.8	12.1	10.5	11.6	0.90	0.34	*0.61*	0.02	0.38
PC aa C40:5	16.6	19.6	16.8^b^	17.3^b^	20.3^a^	1.37	0.17	*0.32*	< 0.01	0.33
PC aa C40:6	3.68	4.50	3.80	4.06	4.42	0.37	0.05	*0.25*	0.30	0.69
PC aa C42:1	0.17	0.18	0.20^y^	0.17^z^	0.17	0.01	0.38	*0.53*	0.02	0.28
PC aa C42:4	0.29	0.26	0.30^a^	0.24^b,z^	0.29^y^	0.02	0.26	*0.55*	0.01	0.27
PC aa C42:5	1.15	1.21	1.45^a^	1.09^b^	1.01^b^	0.13	0.44	*0.56*	< 0.01	0.87
PC aa C42:6	0.49	0.58	0.60^a^	0.52^b,y^	0.48^b,z^	0.02	0.00	*0.01*	< 0.01	0.19

^ab^Indicate differences among LS means within phase at *P* ≤ 0.05 after FDR correction

^yz^Indicate differences among LS means within phase when *0.05* < *P ≤ 0.10* after FDR correction

All significant sphingomyelins, cholesteryl esters, ceramides and fatty acids were found in higher concentrations in MP cows. TG18:0_30:0 (*P* = 0.01) was found at higher concentrations in PP cows. Lysophosphatidylcholines C16:0 (*P* = 0.01), C16:1 (*P* < 0.01), C18:1 (*P* = 0.04), C18:2 (*P* = 0.01) were decreased in PP cows, with the exception of C17:0 (*P* = 0.09) which showed a trend of increase. Phosphatidylcholines PC ae C34:2 (*P* = 0.02), PC aa C28:1 (*P* = 0.02), PC aa C34:2 (*P* = 0.02) are higher in MP cows, whilst phosphatidylcholines PC ae C36:0 (*P* = 0.01), PC aa C36:1 (*P* = 0.00) and PC aa C42:6 (*P* = 0.00) are higher in naiver cows.

Pathway enrichment analysis (Fig. 3a) showed that oxidation of branched chain fatty acids, mitochondrial beta-oxidation of long chain fatty acids and carnitine synthesis were significantly enriched in MP cows when compared with PP cows. Network analysis (Fig. 3b) was constructed in order to visualize the pathway impact in the differences between parities. The most notable pathways were mainly associated with amino acid metabolism.

### Metabolic profile of cows fed moderate versus high-grain

**Fig. 2:**
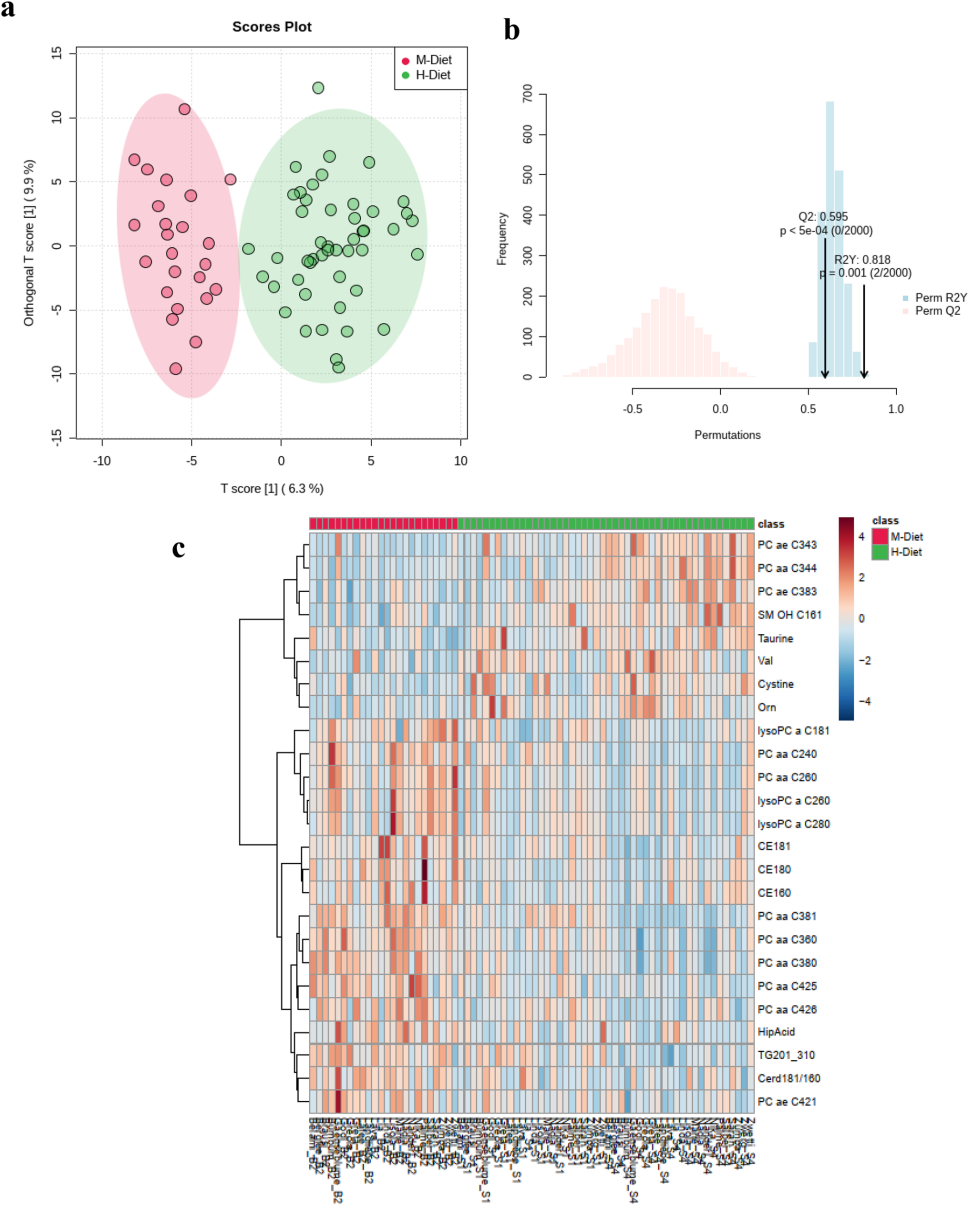
Orthogonal-orthogonal projections to latent structures discriminant analysis (OPLS-DA) showing the cluster separation between M- and H-diets (**a**). Model validation was performed using a permutation test (**b**). Heatmap of the most important variables contributing to the difference observed in the serum metabolome during M-diet and H-diet (**c**)

PCA and OPLS-DA analysis of 393 metabolites that were quantified in 72 serum samples were used as clustering tools to identify the metabolites contributing to the discrimination between cows fed moderate versus high-grain diets (Figs. S2 and 2a, respectively). Cross validation of the OPLS-DA model revealed a Q^2^ value with significant cross-validated values between parity groups (Q^2^ = 0.595, R^2^Y = 0.818 and permutation test *P*-value < 0.00 for 2000 permutations) (Fig. 2b). The dendrogram in Fig. 2c shows the presence of two main clusters corresponding to metabolites that increase or decrease with H-diet. For simplification, only the top 25 metabolites are depicted. The behaviour of each variable according to the diet is indicated with changes in the color intensity on the heatmap. Amino acid valine and amino acid related metabolites cystine and taurine were classified as important features. Phosphatidylcholines PC aa C36:0, PC aa C38:1, PC aa C38:0, PC aa 26:0, PC aa C42:5, PC aa C24:0, PC ae C34:3, PC ae C38:3 and PC aa C42:6 were crucial for profile separation between M-diet and L-diet. The cholesteryl esters CE 18:0 and CE 18:1 decreased during high-grain challenge, being consistent with the overall decrease of this class of metabolites in H-wk1 and H-wk4. Lysophosphatidylcholine lysoPC a C26:0 decreased throughout H-diet. Between the second week of the M diet and the first week of the H diet, there was an overall decrease in the concentrations of carboxylic acids, cholesteryl esters, ceramides, diglycerides and lysophosphatidylcholines (Tables 1, 2 and 3). All the other classes of metabolites increased during this period. The most impacted metabolites were the bile acids cholic acid, chenodeoxylic acid and deoxycholic acid, which increased by 246% (*P* = 0.00), 373% (*P* < 0.01) and 216% (*P* = 0.00), respectively, during the H diet. On the contrary, taurodeoxycholic acid (*P* = 0.00) decreased. Acylcarnitines increased by 8%, particularly due to C18:1 (*P* = 0.00), C3 (*P* = 0.01), C8 (*P* < 0.01) and C0 (*P* < 0.01). A trend between phase and parity was found for several acylcarnitines.

All detected amino acids increased during this period, particularly cysteine, valine and leucine (*P* < 0.01). Cows fed H diets had a lower amount of amino acid related compounds such as *trans*-4-hydroxyproline (*P* < 0.01) and *cis*-4-hydroxyproline (*P* < 0.01). Taurine (*P* < 0.01), ornithine (*P* < 0.01), cystine (*P* < 0.01), homoarginine (*P* = 0.00) and 5-aminovaleric acid (*P* < 0.01), which increased more than 20% in H-wk1. The opposite behaviour was exhibited by 1-methylhistidine (*P* < 0.01) which decreased during this phase. Concentrations of p-Cresol in serum increased by 16% (*P* = 0.00).

Hippuric acid was significantly affected by the feeding phase, decreasing 25% in H-wk1 (*P* = 0.008). The concentration of indoxyl sulfate (*P* = 0.014) increased by 9% in H-wk1 and was 25% higher in H-wk4 when compared with M-diet.

All detectable cholesteryl esters were decreased in H-wk1, except CE 18:2 (*P* < 0.01) and CE 20:3 (*P* < 0.01), which increased up to 28% and 51% in H-wk4, when compared with M. Sphingomyelins (OH) C14:1 (*P* < 0.01), (OH) C16:1 (*P* < 0.01), C16:1 (*P* < 0.01) increased during Lwk1, whilst C24:0 (*P* = 0.01) and C24:1 (*P* = 0.02) decreased. Palmitic acid (P = 0.07) exhibited a trend of decrease while eicosatrienoic acid increased (*P* = 0.06). Overall triglycerides concentration increased by 84% in H-wk1, with the vast majority of the metabolites belonging to this class being overproduced during this phase. TG18:1_34:2 (*P* = 0.01), TG18:2_32:1 (*P* = 0.00) and TG18:3_36:2 (*P* = 0.00) were notorious for their increase, while TG20:1_24:3 (*P* = 0.04) and TG 20:1_31:0 (*P* < 0.01) decreased during H-wk1. All lysophosphatidylcholines decreased, with exception of PC a C17:0 (*P* = 0.00). The same was observed for all ceramides and derivatives. The phosphatidylcholines concentration was the highest in H-wk4, mainly boosted by the increase of PC aa C34:4, PC aa C34:2, PC aa C32:3, PC aa C36:4, PC aa C36:6, PC aa C38:3, PC ae C34:3, PC ae C36:2, PC ae C36:4, PC ae C36:5, PC ae C38:3, PC ae C40:5, PC ae C40:4, PC ae C38:6 and PC ae C38:5 (*P* < 0.01). On the contrary, the lowest concentrations of PC aa C42:5, PC aa C38:1, PC aa C38:0, PC aa C24:0 and PC aa C26:0 (*P* < 0.01) were registered in H-wk4.

**Fig. 3:**
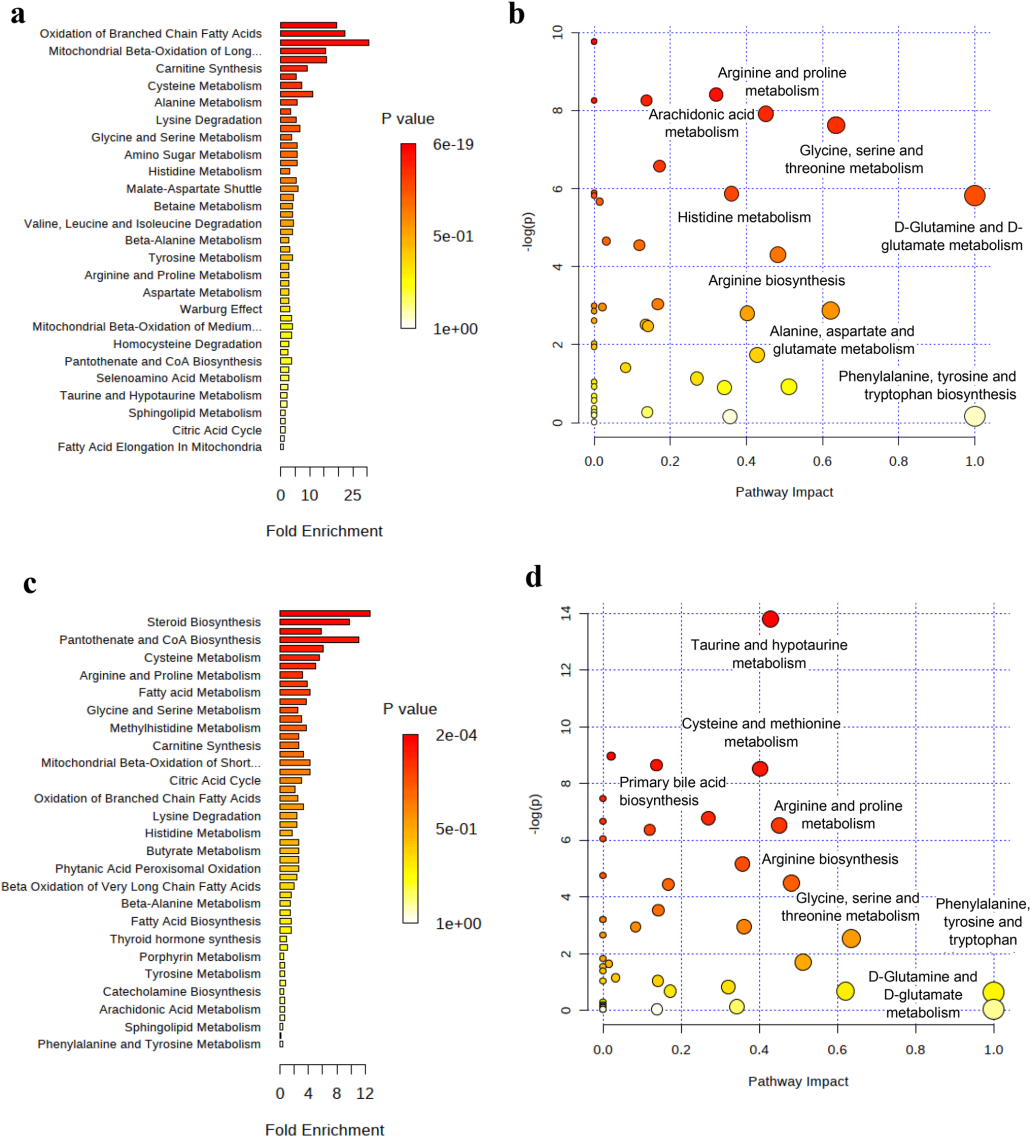
Plot summarizing the meaningful metabolic pathways in serum from the metabolite sets enrichment analysis (MSEA) and pathway analysis according to parity (**a**, **b**) and diet (**c**, **d**), respectively

Pathway enrichment (Fig. 3c) analysis highlighted the enrichment of cellular functions related with steroid biosynthesis, pantothenate and CoA biosynthesis, cysteine metabolism, arginine and proline metabolism as the most significant pathways involved in the dietary switch. Network analysis further revealed the most enriched pathways (Fig. 3d). During high-grain challenge, amino acid, taurine and hypotaurine, and primary bile acids metabolism have the greatest impact in the metabolic response.

## Discussion

Rumen acidosis has evolved to become a main concern in dairy cattle threatening animal health and welfare. This study primarily aimed to establish a relationship between changes in metabolic fingerprinting occurring in dairy cows of different parities during early lactation and high-grain feeding and to understand how ruminal acidosis is reflected in the serum metabolome in lactating cows. We hypothesized that the grain-rich challenge and the resulting ruminal acidosis will lead to impaired energy and lipid metabolism, and this effect will be greater in the primiparous cows. For this, this study surveyed 393 serum metabolites of primiparous and multiparous dairy cows during medium- (M diet) and high-grain (H diet) feeding. This metabolome analysis was part of a broader study (Stauder et al. 2020), in which cows fed H diets showed an impaired rumination activity and increased risk of rumen acidosis, especially during the first week (H-wk1). This impaired metabolic status was accompanied by milk fat depression and increased concentration of liver enzymes in the serum (Stauder et al. 2020). Additionally, PP cows had a higher risk of developing rumen acidosis, resulting in an increased impairment of the liver health variables compared with MP cows.

Previous research has already established that rumen acidosis, caused by starchy concentrates, leads to the production of biogenic amines which are mainly produced in ruminants from the decarboxylation of arginine, lysine, and arginine/ornithine (Saleem et al. 2012). The same research showed previously an increase of several toxic and inflammatory compounds in the rumen and interference with amino acid biosynthesis, particularly phenylalanine, ornithine, lysine, leucine, arginine, valine, and phenylacetylglycine in early-lactating primiparous dairy (Saleem et al. 2012).

In our study, the PP cows showed higher concentrations of *trans*-4-hydroxyproline and *cis*-4-hydroxyproline compared to MP cows. These observations were previously reported to be associated with lipid mobilization in early postpartum cows, where primiparous cows showed higher concentrations of serine, methionine-sulfoxide and *trans*-4-hydroxyproline compared to multiparous cows (Humer et al. 2016a). The latter metabolite was identified as a significant metabolite on day 21 after parturition in normal and over-conditioned cows (Ghaffari et al. 2019). Both metabolites were also decreased in cows experiencing ruminal acidosis, particularly at H-wk1. A decrease in *trans*-4-hydroxyproline in cows experiencing ruminal acidosis has been previously reported (Humer et al. 2018a). Although hydroxyproline has been traditionally considered to have little nutritional significance, it is now recognized as a substrate for the synthesis of glycine, pyruvate, and glucose (Wu et al. 2011). The hydroxylation of proline may also scavenge oxidants and regulate the redox state of cells (Phang et al. 2008, 2010). It is possible that hydroxyproline may spare proline by reducing proline catabolism or stimulate tissue protein synthesis through multiple signalling pathways. These amino acid related compounds also decreased throughout high grain feeding (H-wk1), which is again in concordance with previous results reported in cows having ruminal acidosis (Humer et al. 2018b). Glutamate is an important amino acid and plays a key role in amino acid metabolism by providing amino groups for the formation of other amino acids via transamination or deamination of 2-ketoglutaric acid. Glutamate and glutamine degradation in enterocytes yields products such as proline, ornithine, citrulline, arginine, and alanine (Wu et al. 1995). A significant increase in proline, ornithine and arginine between M and H-wk4 was observed. Glutamate decreased between H-wk1 and H-wk4. PP cows were found to have increased levels of glutamate when compared to MP cows. Additionally, proline betaine was identified as a significant feature both using MV analysis approaches. MP cows showed a higher serum content of proline betaine when compared with PP cows. Recent findings suggest that proline may play a role in regulating the mammalian target of rapamycin (mTOR) activation pathway (Van Meijl et al. 2010), which integrates signals from nutrients such as glucose and amino acids, cellular energy status, growth factors, and various stress factors to affect cell growth and function (Li et al. 2009; Liao et al. 2008). Proline, together with arginine, glutamine and leucine enhances protein synthesis in cells and tissues and polyamine synthesis via proline oxidase and ornithine decarboxylase (Wu et al. 2010). Glycine and serine were previously shown to decrease during bouts of rumen acidosis, whereas biogenic amines carnosine and taurine increased (Humer et al. 2018a, b). Biogenic amines have been suggested as a biomarker of bacterial dysbiosis during rumen acidosis in cattle (Plaizier et al. 2012). In our study, taurine and cystine were both shown to increase significantly during high-grain feeding (H-wk1 and H-wk4, respectively). While serine and glycine were identified as significant amino acids regarding cow parity, valine was identified as a significant metabolite in H-diet by MV analysis. All significant amino acids were shown to increase after switching from M- to the H- diet. Cereal grains are rich in starch, which is rapidly degraded in the rumen and releases large amounts of volatile fatty acids, including acetate, propionate, and butyrate, as well as other organic acids such as lactate (Iqbal et al. 2009). Only a slight increase in carboxylic acids was observed, whereas hippuric acid decreased with grain feeding, which can be explained with the decrease of forage level in the diet (Carpio et al. 2013). The steroid acids cholic acid, chenodeoxylic acid and deoxycholic acid greatly increased in the serum during H-wk1, whilst conjugated bile acids like glycocholic and taurocholic acid rather decreased. The primary bile acids such as cholic and chenodeoxycholic acids are produced in the liver from the catabolism of cholesterol in cattle (Sheriha et al. 1968). The cholesterol indeed significantly decreased in the blood of cows during high grain feeding in this research (Stauder et al. 2020), indicating that an increased cholesterol catabolism led to an increased synthesis of primary bile acids during high grain feeding. Primary bile acids are then conjugated with either glycine or taurine to produce glycocholic and taurocholic acid at the expense of cholyl CoA and assist fat digestion and absorption in the small intestine of cattle (Sheriha et al. 1968). Conjugation is known to happen in the liver and in the gut by the activity gut microbes, whereby from other species is known that the diet affects the bile acid conjugation via a modulation of the microbiome activity (Ghaffarzadegan et al. 2019). The exact mechanism behind a decrease of conjugated bile acids and the increase of their precursors with high grain feeding is not clear but may indicate a decreased conjugation most likely in the gut but also in the liver. Further unpublished results of our study indicate a hindgut dysbiosis in cows fed high grain diet, whereas a liver tissue damage was also evident and reported in the companion paper (Stauder et al. 2020). On the other hand, our network analysis revealed that one of the most enriched pathways involved the taurine, hypotaurine, and primary bile acids metabolism as having the greatest impact in the metabolic response during the high-grain challenge, indicating an accumulation of the primary bile products in the blood during high-grain feeding. Our data suggest an increased ratio between the primary bile acids and conjugated bile acids in the blood to be a good indicator of the subclinical metabolic disturbances related to grain-rich feeding.

Acylcarnitines or their overall profiles were previously suggested as novel biomarkers for lipid mobilization in dairy cows (Humer et al. 2016a). Primiparous cows had higher levels of carnitine and acylcarnitines when compared with MP cows. These results are in accordance with previous observations from (Humer et al. 2016a), where the concentrations of acylcarnitines C0, C2, C3, C4 and C5 were decreased in multiparous compared to primiparous cows. Given the fundamental role of carnitine in hepatic fatty acid oxidation (Bremer 1983), the authors suggested that the carnitine status might influence the degree of liver lipid accumulation in peripaturient dairy cows. Since carnitines mediate the transport of long-chain fatty acids from the cytosol into the mitochondria of hepatocytes (Longo et al. 2006), reduced concentrations of free carnitine in MP cows seem to reflect an enhanced need of carnitine for the transport of fatty acids due to the higher mobilization from the stored lipids to generate metabolic energy in these cows. Enhanced levels of acetylcarnitine and decreased levels of propionylcarnitine concentrations were previously reported in association with incomplete LCFA ß-oxidation in human type 2 diabetes (Adams et al. 2009). Previous research suggested an increased risk for high lipid mobilization in MP cows (Humer et al. 2016a,b). Lysophosphatidylcholine C26:0 was increased during the M-diet (*P* = 0.0003) and was identified by MV analysis as a significant feature in cluster separation between diets. Elevated levels of this lysophosphatidylcholine were previously reported in human patients with Zellweger spectrum disorders, a group of metabolic disorders caused by a genetically encoded defect in peroxisome biogenesis (Klouwer et al. 2017), and in X-linked adrenoleukodystrophy, a progressive neurodegenerative disorder (Huffnagel et al. 2017). Both studies showed that lyso PC C26:0 was a sensitive marker for the accumulation of very long-chain fatty acids in plasma due to a deficient peroxisomal beta-oxidation of these FA (Huffnagel et al. 2017; Klouwer et al. 2017). A previous study in dairy cows identified a decrease in the concentrations of phosphatidylcholines, lysophosphatidylcholines, sphingomyelines, and several AA in the blood during the first bout of rumen acidosis (Humer et al. 2018a). However, despite an evident decrease in the concentration of lysoPC during H-wk1, this study indicates a slight increase in PC during H-wk1, and at Lwk4 the overall concentration of PC and lysoPC was higher than during M feeding. Cows receiving lipopolysaccharides from *E. coli* (O26:B6) intramammarily or experiencing diseases were previously reported to have lower levels of plasma lysoPC (Hailemariam et al. 2014a, b; Humer et al. 2018b). The most significant changes in the blood metabolome of dairy cows during the first months of lactation were mainly associated with the levels of polyunsaturated fatty acids containing phosphatidylcholine (Ilves et al. 2012). MP cows had overall increased concentrations in serum of lysophosphatidylcholines, phosphatidylcholines, ceramides, triglycerides, sphingomyelins and fatty acids when compared to PP cows. Lysophosphatidylcholine C16:1 (*P* < 0.001) was more often found in MP than PP cows at it was previously reported in association with birth weight in humans, with lower birth weight newborns having lower serum concentrations of this lysoPC (Lu et al. 2018).

## Conclusion

Our data showed major differences in the metabolomics responses of PP and MP cows to a high grain diet challenge. PP cows had an overall 40% increase in the serum levels of acylcarnitines and some phosphatidylcholines (e.g., PC ae C36:0, PC aa C36:1, PC aa C42:6) when compared with MP cows but lower serum concentrations of sphingomyelins, cholesteryl esters, ceramides and fatty acids as well as of most lysophosphatidylcholines (e.g., C16:0, C16:1, C18:1, C18:2). Given these results, more parity-shaped feeding and management strategies for dairy cows in the future is highly recommended. Our study also revealed that increasing grain level in the diet from 40 to 60% decreased the concentrations of carboxylic acids, cholesteryl esters, ceramides, diglycerides and lysophosphatidylcholines, but increased the accumulation of primary bile acids by over 250% and decreased the conjugated bile acids, likely due to the gut dysbiosis. In addition, cows experiencing higher rumen acidosis had major alterations in the serum concentrations of amino acids and amino acid related compounds such as trans-4-hydroxyproline and cis-4-hydroxyproline, taurine, ornithine, cystine, homoarginine, and 5-aminovaleric acid. Such metabolomics fingerprints clearly distinguishing moderate and high-grain feeding hold potential of early diagnostic tools for cows experiencing grain-induced metabolic disturbances.

## Electronic supplementary material

Below is the link to the electronic supplementary material.

Supplementary file1 (DOCX 115 kb)

## Data Availability

All relevant data are within the article and its Supplementary info. The metabolomics and metadata reported in this paper are available via Metabolomics Workbench (https://www.metabolomicsworkbench.org/) study identifier ID ST001349.
