# Supplementary material for: Distinct serum metabolomic signatures of multiparous and primiparous dairy cows switched from a moderate to high-grain diet during early lactation
Source: Metabolomics. 2020 Sep 9;16(9):96. doi: 10.1007/s11306-020-01712-z (PMC7481167; doi:10.1007/s11306-020-01712-z)
Supplement: Supplementary file 1 — Supplementary file1 (DOCX 115 kb) [file 11306_2020_1712_MOESM1_ESM.docx]

**Table S1.** Data on parity, weight, milk yield, DMI and Acidosis index for each cow enrolled in this study during moderate grain feeding (M) and high grain feeding during week 1 (H-wk1) and 4 (H-wk4).

| **Parity** | **Cow number** | **Weight (kg)** | | **DMI (kg/d)** | | | **Milk yield (kg/d)** | | | **Acidosis index^1^** | | |
| --- | --- | --- | --- | --- | --- | --- | --- | --- | --- | --- | --- | --- |
|  |  | **Initial** | **Final** | **M** | **H-wk1** | **H-wk4** | **M** | **H-wk1** | **H-wk4** | **M** | **H-wk1** | **H-wk4** |
| PP | 21 | 630 | 674 | 19.0 | 20.9 | 36.1 | 29.3 | 31.8 | 32.5 | 18.5 | 20.2 | 15.6 |
| PP | 23 | 664 | 720 | 19.0 | 22.8 | 23.9 | 33.2 | 35.1 | 33.5 | 1.36 | 1.09 | 0.00 |
| PP | 2 | 614 | 666 | 21.3 | 19.9 | 22.7 | 33.9 | 34.1 | 34.3 | 9.33 | 13.0 | 16.6 |
| PP | 11 | 594 | 678 | 16.5 | 19.8 | 25.6 | 26.8 | 28.1 | 31.7 | 31.2 | 28.6 | 28.9 |
| PP | 8 | 616 | 636 | 17.1 | 18.2 | 16.8 | 28.2 | 27.5 | 28.9 | 36.0 | 41.7 | 34.4 |
| PP | 14 | 658 | 720 | 20.5 | 20.5 | 23.9 | 34.0 | 35.9 | 38.3 | 2.07 | 11.8 | 2.05 |
| PP | 16 | 632 | 682 | 19.1 | 18.3 | 21.6 | 31.0 | 32.2 | 35.0 | 17.0 | 24.9 | 15.6 |
| PP | 17 | 566 | 636 | 16.9 | 20.3 | 22.7 | 32.8 | 34.8 | 40.5 | 0.06 | 1.24 | 0.00 |
| MP | 1 | 746 | 780 | 19.5 | 19.6 | 21.3 | 40.1 | 39.9 | 42.3 | 3.24 | 9.44 | 9.44 |
| MP | 6 | 690 | 748 | 20.4 | 24.8 | 27.1 | 39.2 | 42.4 | 41.8 | 17.0 | 12.2 | 7.34 |
| MP | 22 | 890 | 890 | 22.4 | 22.6 | 22.2 | 30.5 | 34.0 | 32.5 | 11.6 | 12.8 | 9.30 |
| MP | 7 | 792 | 838 | 22.8 | 25.6 | 21.7 | 44.5 | 44.3 | 45.2 | 3.93 | 16.1 | 1.92 |
| MP | 12 | 994 | 1025 | 18.2 | 18.5 | 23.8 | 34.7 | 36.2 | 40.5 | 0.00 | 0.00 | 0.00 |
| MP | 9 | 670 | 690 | 21.1 | 23.2 | 28.9 | 45.5 | 46.9 | 49.9 | 3.74 | 3.70 | 2.45 |
| MP | 24 | 764 | 816 | 20.2 | 25.5 | 30.8 | 35.4 | 39.8 | 41.1 | 13.0 | 11.2 | 2.87 |
| MP | 3 | 772 | 826 | 21.6 | 22.3 | 22.6 | 41.0 | 40.5 | 39.6 | 27.5 | 32.0 | 31.1 |
| MP | 13 | 686 | 718 | 17.6 | 18.8 | 18.5 | 31.6 | 32.7 | 33.2 | 14.9 | 9.60 | 7.98 |
| MP | 18 | 734 | 812 | 23.9 | 20.7 | 24.7 | 39.1 | 44.8 | 51.8 | 0.00 | 0.13 | 0.00 |
| MP | 19 | 721 | 754 | 20.5 | 19.7 | 20.2 | 36.0 | 36.8 | 35.6 | 4.94 | 20.8 | 4.67 |
| MP | 20 | 750 | 810 | 19.8 | 21.4 | 26.4 | 35.7 | 36.4 | 37.5 | 0.74 | 3.21 | 0.00 |
| MP | 4 | 668 | 742 | 30.1 | 23.2 | 25.2 | 40.3 | 39.9 | 40.3 | 0.00 | 0.00 | 0.00 |
| MP | 10 | 760 | 842 | 21.8 | 26.6 | 22.1 | 40.6 | 44.7 | 44.8 | 12.2 | 0.65 | 3.73 |
| MP | 5 | 714 | 866 | 25.1 | 24.8 | 23.2 | 44.7 | 50.7 | 51.3 | 0.00 | 0.00 | 0.00 |
| MP | 15 | 762 | 750 | 24.6 | 23.6 | 25.1 | 42.0 | 44.7 | 51.8 | 4.55 | 3.45 | 0.00 |

^1^ruminal acidosis index which is the area of pH below 5.8 divided by DMI (pH x min/kg); the higher the index, the higher the risk of the cows to develop a subacute rumen acidosis.

**Table S2.** Ingredients, nutrient composition and particle size distribution of the moderate-grain diet (M-diet) or the diet with high-grain (H-diet).

| **Item** | **M-diet** | **H-diet** |
| --- | --- | --- |
| Ingredient (% of dry matter) |  |  |
| Grass silage | 30.0 | 20.0 |
| Corn silage | 30.0 | 20.0 |
| Barley grain | 25.2 | 37.8 |
| Soybean meal | 6.0 | 9.0 |
| Corn grain | 3.6 | 5.4 |
| Rapeseed meal | 3.2 | 4.8 |
| Mineral-vitamin premix^1^ | 0.8 | 1.2 |
| Limestone  Beet pulp | 0.48  0.4 | 0.72  0.6 |
| Monocalcium phosphate | 0.2 | 0.3 |
| Sodium chloride | 0.12 | 0.18 |
| Nutrient composition | | |
| DM, % of fresh matter | 47.6 | 50.2 |
| Organic matter | 93.0 | 93.0 |
| Crude protein | 16.5 | 18.2 |
| Ether extract  Neutral detergent fiber (NDF) | 1.94  33.2 | 1.94  29.4 |
| Acid detergent fiber (ADF) | 20.8 | 17.3 |
| Starch | 26.3 | 32.0 |

^1^The mineral-vitamin premix contained 13.5% Calcium, 9% Magnesium, 5% Phosphorus, 1.5% Sodium, 1,800,000 IU vitamin A/kg, 300,00 IU vitamin D/kg, 7,500 mg vitamin E/kg, 70 mg vitamin B1/kg, 180 mg vitamin B2/kg, 145 mg vitamin B6/kg, 1,800 µg vitamin B12/kg, 1,800 mg niacin/kg, 305 mg pantothenic acid/kg, 36 mg folate/kg, 11,800 mg choline/kg, 13,500 mg manganese(II) oxide/kg, 19,800 mg zinc oxide/kg, 4,500 mg copper(II) sulfate/kg, 450 mg iodine (calcium iodine)/kg, 120 mg selene (sodium selenite)/kg, and 195 mg cobalt(II) carbonate/kg.

**Figure S1**. Tridimensional score plot between PC1, PC2 and PC3 in regards to cow parity (MP – red, PP –green).

**Figure S2**. Tridimensional score plot between PC1, PC2 and PC3 in regards to feeding phase (M-diet – red, H-diet – green).
